# Exploring the repurposed role of solithromycin as an antivirulence agent against *Staphylococcus aureus* and its resistant variants

**DOI:** 10.3389/fmicb.2025.1540997

**Published:** 2025-03-27

**Authors:** Rihaf Alfaraj, Fai A. Alkathiri, Lama A. Alamri, Najd B. Alnassar, Sarah H. Alanazi, Razan A. Algarni, Norah S. Alhabdan, Reema A. Abuthnain

**Affiliations:** Department of Pharmaceutics, College of Pharmacy, King Saud University, Riyadh, Saudi Arabia

**Keywords:** *Staphylococcus aureus*, solithromycin, MRSA, *ΔagrA*, antivirulence, biofilm inhibition

## Abstract

**Introduction:**

*Staphylococcus aureus* is a bacterium that can cause various infections. The rise in Staphylococcal-resistant infections has led to the need for new treatments. The accessory gene regulator (*agr*) quorum-sensing system, which regulates the expression of genes involved in hemolysin, protease, and biofilm production, has been implicated in the virulence of *S. aureus’s* pathological characteristics.

**Objectives:**

This study investigates the potential of Solithromycin (SOL), a next-generation macrolide with broad-spectrum activity, to be repurposed as an antivirulence agent against *S. aureus*, MRSA, and *ΔagrA* strains.

**Methods:**

Using various antibacterial assays, the antibacterial and antivirulence activities of SOL were evaluated against *S. aureus*, MRSA, and *ΔagrA* strains. The sub-inhibitory concentration MIC_50_ of SOL was tested for anti-virulence activity by assessing motility, biofilm formation, hemolysin, and protease production. Scanning electron microscopy (SEM) and confocal laser scanning microscopy (CLSM) were used to visualize biofilm morphology. Conventional PCR was used to detect virulence genes following SOL treatment.

**Results:**

SOL demonstrated significant antibacterial efficacy against *S. aureus*, MRSA, and *ΔagrA* strains with MIC_90_ (0.8 μg/mL) and MIC_50_ (0.4 μg/mL). SOL decreased *S. aureus* motility at MIC_50_ but had no effect on MRSA and *ΔagrA* strains. Hemolysin and protease activities were unaffected in all the tested strains. SEM and CLSM revealed significant reductions in biofilm formation and thickness. SOL at MIC_90_ and MIC_50_ reduced Congo red staining intensity. MIC_50_ inhibited MRSA and *ΔagrA* biofilms by 36.6 and 56.4%, respectively, with no significant effect on *S. aureus* biofilms in the crystal violet assay. PCR showed no leukocidin gene in the treated strains.

**Discussion:**

This study highlights the potential of SOL as an antivirulence agent, emphasizing the importance of targeting regulators, such as *ΔagrA*, in managing *S. aureus* infections.

## 1 Introduction

*Staphylococcus aureus* remains a significant global health concern, causing a wide spectrum of infections, ranging from superficial skin lesions to life-threatening systemic diseases (Goc et al., 2023). The emergence and spread of methicillin-resistant *S. aureus* (MRSA) strains have further complicated treatment strategies, leading to increased morbidity, mortality, and healthcare costs (Liu et al., 2015). This antibiotic resistance crisis has necessitated the exploration of novel therapeutic approaches, including targeting bacterial virulence mechanisms rather than solely focusing on bacterial growth inhibition. One promising target for antivirulence strategies is the accessory gene regulator (agr) quorum-sensing system in *S. aureus* (Goc et al., 2023). The agr system plays a crucial role in regulating the expression of various virulence factors, including toxins, proteases, and other factors involved in biofilm formation. AgrA protein, a response regulator within this system, is vital for the activation of virulent gene expression. Targeting the agr system or its components may attenuate bacterial virulence without imposing a strong selective pressure for resistance development (Rodgers et al., 2013).

Macrolides have shown promising potential as antivirulence agents against various bacterial pathogens, including *S. aureus* (Tsakou et al., 2020). These antibiotics can interfere with bacterial quorum-sensing systems, inhibit biofilm formation, and modulate virulence factor production at sub-inhibitory concentrations (Udekwu et al., 2009). However, macrolides face limitations, such as increased bacterial resistance and potential side effects with long-term use (Vandenesch et al., 2012). Solithromycin (SOL), a next-generation ketolide, offers several advantages over traditional macrolides that could enhance its antivirulence potential. SOL, a next-generation macrolide antibiotic, has shown promising antibacterial activity against *S. aureus*, including methicillin-resistant *S. aureus* (MRSA) strains (Amábile-Cuevas, 2023). Previous studies have shown its potent *in vitro* efficacy, with MIC_90_ values as low as 0.06 μg/mL for MRSA, outperforming other macrolides. Notably, SOL has maintained activity against *S. aureus* isolates resistant to erythromycin and azithromycin, suggesting its potential for combating antibiotic-resistant strains (Putnam et al., 2010). Although its antibacterial properties are well documented, there remains a significant gap in our understanding of the potential antiviral effects of SOL against *S. aureus* and MRSA. Limited information exists on its impact on virulence factors, biofilm formation, and underlying mechanisms of action in this context. Furthermore, the long-term implications of resistance development and efficacy in various clinical settings require further investigation. This study aimed to address these knowledge gaps by exploring the role of SOL as an antivirulence agent and assessing the impact of SOL on bacterial virulence factors, including motility, biofilm formation, hemolysin production, and protease activity. The effects of SOL on the biofilm structure and morphology were investigated using advanced microscopy techniques. The molecular mechanisms underlying SOL antivirulence activity were examined using traditional molecular detection.

## 2 Materials and methods

### 2.1 Bacterial strains, chemicals, and growth conditions

All bacterial strains listed in Table 1, are obtained from American Type Culture Collection (ATCC). Bacteria and mutant obtained from the library of MRSA transposon from the University of Washington Genome Centre (Washington University, Seattle, WA, USA), and stored at −80°C as glycerol stocks. For the experiments the bacterial strains were grown at 37°C overnight in a shaking incubator (200 rpm). The *ΔagrA* strain was supplemented with 15 μg/mL erythromycin as a selective marker. All cultural media, including LB agar, blood agar plates, Congo red, and brain heart infusion agar with skimmed milk (Thermo Fisher, Waltham, MA, USA), were prepared according to the manufacturer’s instructions.

**Table 1:** Bacterial strains used in the study.

Strain name	Abbreviation	Description
*Staphylococcus aureus*	*S. aureus*	Wild type (ATCC 25923)
Methicillin-Resistant *Staphylococcus aureus*	MRSA	Methicillin-Resistant *Staphylococcus aureus* (ATCC 43300)
NE1532	*ΔagrA*	4 P16 agrA accessory gene regulator protein A SAUSA300_1992

The stock solution of SOL was prepared by dissolving 8.5 mg of SOL in 10 mL dimethyl sulfoxide (DMSO) to make a concentration of 1 mg/mL and stored at 4°C. The primer sequences used for PCR are listed in Table 2.

**Table 2:** Primers used in the study (Lin et al., 2021).

Primer	Oligonucleotides sequence 5′–3′	Description/reference
spIB	-F:GAACAAAAACGTAGTCATCAAGAGTTTAGCAGC -R:CTATGTTTTCTGCAATGAATTTTTTAATTTCTGGTGT	Invasive protease (723 bp)
LukF	-F:ATGAAAAAAATAGTCAAATCATCAGTTGTTACATCA -R:AGCTCATAGGATTTTTTTCCTTAGATTGAG	Leukocidin (978 bp)
hla	-F:ATGAAAACACGTATAGTCAGCTCAGTAACAAC -R:TTAATTTGTCATTTCTTCTTTTTCCCAATCGA	Hemolysin (960 bp)

### 2.2 Antibacterial assays

#### 2.2.1 Agar diffusion test

All bacterial strains were grown overnight in LB broth. The following day, the culture was adjusted to 0.5 McFarland standard. Approximately 100 μL of each bacterial suspension was spread on Mueller-Hinton agar (MHA). Briefly, 6 mm-diameter wells were punched through the MHA plates, and then 100 μL of SOL at a concentration of 1 mg/mL was added to each well. The presence of a clear zone around the well indicated the effectiveness of SOL (Bonev et al., 2008).

#### 2.2.2 Growth curve and minimum inhibitory concentration

All bacterial strains were grown overnight in 5 mL of LB broth, except *ΔagrA*, which was supplemented with erythromycin at a concentration of 15 μg/mL. The following day, the bacterial culture was adjusted to 0.5 McFarland standard and then incubated at 37°C with continuous shaking at 200 rpm. After that, 20 μL of bacterial broth (at OD 0.5 McFarland standard) was added to 100 μL of SOL in a seven-fold serial dilution in 100-well honeycomb microplates. All tests were performed in triplicate, including controls of media only and untreated bacteria. Subsequently, the 100-well plates were placed in a Bioscreen C reader (Growth curves Ltd’s, Helsinki, Finland). Samples were collected and the absorbance was read at 600 nm at 60-min intervals for 20 h (Andrews, 2001).

#### 2.2.3 Colony counting

All bacterial strains were grown overnight in 5 mL of LB broth at 37°C. For the *ΔagrA* strain, the broth was supplemented with 15 μg/mL of erythromycin. The next day, the bacterial culture was adjusted to the 0.5 McFarland standard. A seven-fold serial dilution of SOL, starting from 1 mg/mL, was prepared. For each dilution and bacterial strain, 100 μL of the bacterial broth was inoculated onto LB agar plates. The plates were then incubated for 24 h at 37°C. After incubation, the number of colonies that grew on each plate was counted. MIC (minimum inhibitory concentration) is defined as the lowest concentration of SOL that results in no visible growth, while MBC (minimum bactericidal concentration) is defined as the first dilution (starting from 1 mg/mL) that results in no growth after 24 h of incubation at 37°C (Andrews, 2001; Barnes et al., 2023).

### 2.3 Anti-virulence assays

#### 2.3.1 Hemolysis test

All bacterial strains were adjusted to OD 0.5 McFarland standard and inoculated in LB broth with or without SOL at MIC_90_ (0.8 μg/mL) and MIC_50_ (0.4 μg/mL). After incubating at 37°C for 18 h, the bacterial cultures were streaked on blood agar. The zone of red cell clearance around inoculated bacteria was observed (Hrv et al., 2016).

#### 2.3.2 Protease test

All bacterial strains were adjusted to OD 0.5 McFarland standard and inoculated in LB broth with or without SOL at MIC_90_ (0.8 μg/mL) and MIC_50_ (0.4 μg/mL) after incubating at 37°C for 18 h, the bacterial cultures were streaked on BHI and skimmed milk agar plates. The zone of inhibition around inoculated bacteria was observed (Hrv et al., 2016).

#### 2.3.3 Motility test

Modified spreading motility media were used for motility assessment. All bacterial strains were adjusted to OD 0.5 McFarland standard and inoculated in LB broth with or without SOL at MIC_90_ (0.8 μg/mL) and MIC_50_ (0.4 μg/mL) After incubating at 37°C for 18 h, the plates were inoculated with a sterile cotton swab and incubated at 37°C for 24 h. Motility was assessed by observing the colonies migrating away from the point of inoculation (Palma et al., 2022).

#### 2.3.4 Biofilm formation/inhibition assays

##### 2.3.4.1 Crystal violet assay

All bacterial strains with and without SOL MIC_90_ (0.8 μg/mL) and MIC_50_ (0.4 μg/mL) were grown in 96-well microplates containing LB media for 24 h at 37°C. The bacterial broth was discarded gently, and the microplates were washed twice for 2 min with 200 μL 0.9% normal saline (NS) in the shaker and were then left to dry for 15 min in the oven. The resulting biofilm was stained with 150 μL 1% crystal violet (Sigma-Aldrich, St. Louis, MO, USA) for 15 min in the shaker and was then discarded. Excess stain was washed gently by immersing the microplates in 0.9% normal saline (NS). The remaining biofilm was solubilized by adding 200 μL 80:20 (vol/vol) ethanol to acetone. The microplate was evaluated by a microplate reader (Thermo Fisher) using spectrophotometric absorbance measurements at 595 nm. The formula used to get the percentage of reduction was [(OD (control) − OD (test)/OD (control)) × 100] (Kwasny and Opperman, 2010).

##### 2.3.4.2 Congo red agar test (CRA)

The Congo red agar method allows for the direct analysis of biofilm-producing colonies and the identification of slime-forming strains. All bacterial strains were adjusted to OD 0.5 McFarland and inoculated in LB with or without SOL at MIC_90_ (0.8 μg/mL) and MIC_50_ (0.4 μg/mL). After incubating at 37°C for 18 h, the bacterial culture was inoculated on Congo red agar plates. Biofilm was assessed by the appearance of black colonies on the red agar (Harika et al., 2020).

##### 2.3.4.3 Scanning electron microscope analysis (SEM)

All bacterial strains were adjusted to 0.5 McFarland standard and inoculated in LB broth with or without SOL at MIC_90_ (0.8 μg/mL) and MIC_50_ (0.4 μg/mL). After incubating at 37°C for 18 h, Polyvinyl coverslips (Fisher Scientific) were placed in each well of 6-well plates and then 2 mL LB and diluted culture were added. Biofilms formed on the coverslips over 24 h at 37°C and were then fixed with 2.5% glutaraldehyde in PBS, for 24 h. After three washes with PBS, the coverslips were fixed with 1% Osmium tetroxide in H_2_O and then dehydrated using ethanol at 30 min intervals in a graded series (50–95%) and finally in 100% ethanol. All samples were coated with gold thin film. SEM imaging was in high vacuum mode at 20 kV using an FEI Quanta 400 FEG ESEM/EDAX Genesis X4M (FEI Co., Hillsboro, OR, USA) (Alhede et al., 2012).

##### 2.3.4.4 Confocal laser scanning microscopy (CLSM)

All bacterial strains were adjusted to 0.5 McFarland standard and inoculated in LB with or without SOL at MIC_90_ (0.8 μg/mL) and MIC_50_ (0.4 μg/mL) After incubating at 37°C for 18 h, polyvinyl coverslips (Fisher Scientific, USA) were placed in each well of 6-well plates and then 2 mL LB and diluted culture were added. Biofilms formed on the coverslips over 24 h at 37°C. The antibiofilm potential of SOL was tested on all strains. Biofilm was visualized under a CLSM. Protein-coated coverslips (BSA, Sigma-Aldrich, USA) were used on a 6-well chamber slide, on which biofilms developed. The slides were washed twice with distilled water to remove the planktonic cells. After adding 5 μL premixed dyes of SYTO 9 green fluorescent nucleic acid stain (Thermo Fisher, USA), which stains live cells, and propidium iodide (red fluorescence), which stains dead cells for 15 min in the dark, the structure of the biofilm was examined by a CLSM (Zeiss DMi8; Leica Microsystems) (Alhede et al., 2012).

### 2.4 Molecular assays

#### 2.4.1 DNA extraction

An overnight culture of all bacterial strains with or without SOL at MIC_90_ (0.8 μg/mL) and MIC_50_ (0.4 μg/mL). One milliliter of each bacterial culture was transferred to a 1.5 mL microcentrifuge tube and then centrifuged for 2 min at 8,000 rpm. Supernatant was discarded and pellets were treated with lysis buffer to extract the DNA following the instruction manual of the DNA extraction kit (Roche Diagnostics, Switzerland). The DNA samples were stored at −20°C (Dashti et al., 2009).

#### 2.4.2 Amplification using a thermocycler (PCR)

The primers listed in Table 2 were used to detect the *splB*, *hla*, and *lukF* genes. Each PCR was carried out in a 20-μL (final volume) mixture containing 10 mmol of each primer in DreamTaq green PCR master mix (Thermo Fisher). PCR was performed using the recommended thermal cycling conditions outlined in the Thermo Fisher Scientific manual (Lorenz, 2012).

#### 2.4.3 Agarose gel electrophoresis

The PCR products were detected by running electrophoresis on a 1% agarose gel prepared with 1X Tris-Borate EDTA (TBE) buffer. To prepare the 1% agarose gel, 1 gram of agarose powder (Sigma-Aldrich, USA) was mixed with 150 mL of TBE buffer. The mixture was heated until the agarose was fully dissolved and then cooled to 60–70°C. Next, 0.5 μg/mL of ethidium bromide was added. A comb was placed in a gel tray, and the agarose gel was poured. After the gel solidified, the comb was removed. In one well, 1 μL of 1 Kb DNA ladder was mixed with 1 μL of loading dye and 4 μL of sterile water. In each well, 10 μL of the mixed sample was loaded. The gel, containing the amplicon and DNA ladder (1Kb, Qiagen, DE), was then placed in the electrophoresis tank (Biorad, USA) with 1X TBE buffer for 40 min at 110 V. Finally, the gel was visualized using UV light (Stegger et al., 2012).

### 2.5 Statistical analysis

Statistical analysis was performed in GraphPad Prism 10.4.0 (GraphPad Software, Boston, MA, USA). The Kruskal–Wallis test was employed and then interpreted as ANOVA for multiple comparisons (Kim, 2014).

## 3 Results

### 3.1 Antibacterial assays

#### 3.1.1 Agar diffusion test

The agar diffusion test showed clear zones of inhibition around the wells containing SOL, indicating positive antibacterial activity. The bacterial strains tested demonstrated varying degrees of susceptibility to SOL. A distinct clear zone, measuring approximately 20–25 mm in diameter, was observed.

#### 3.1.2 Growth curve and minimum inhibitory concentration

**Figure 1:**
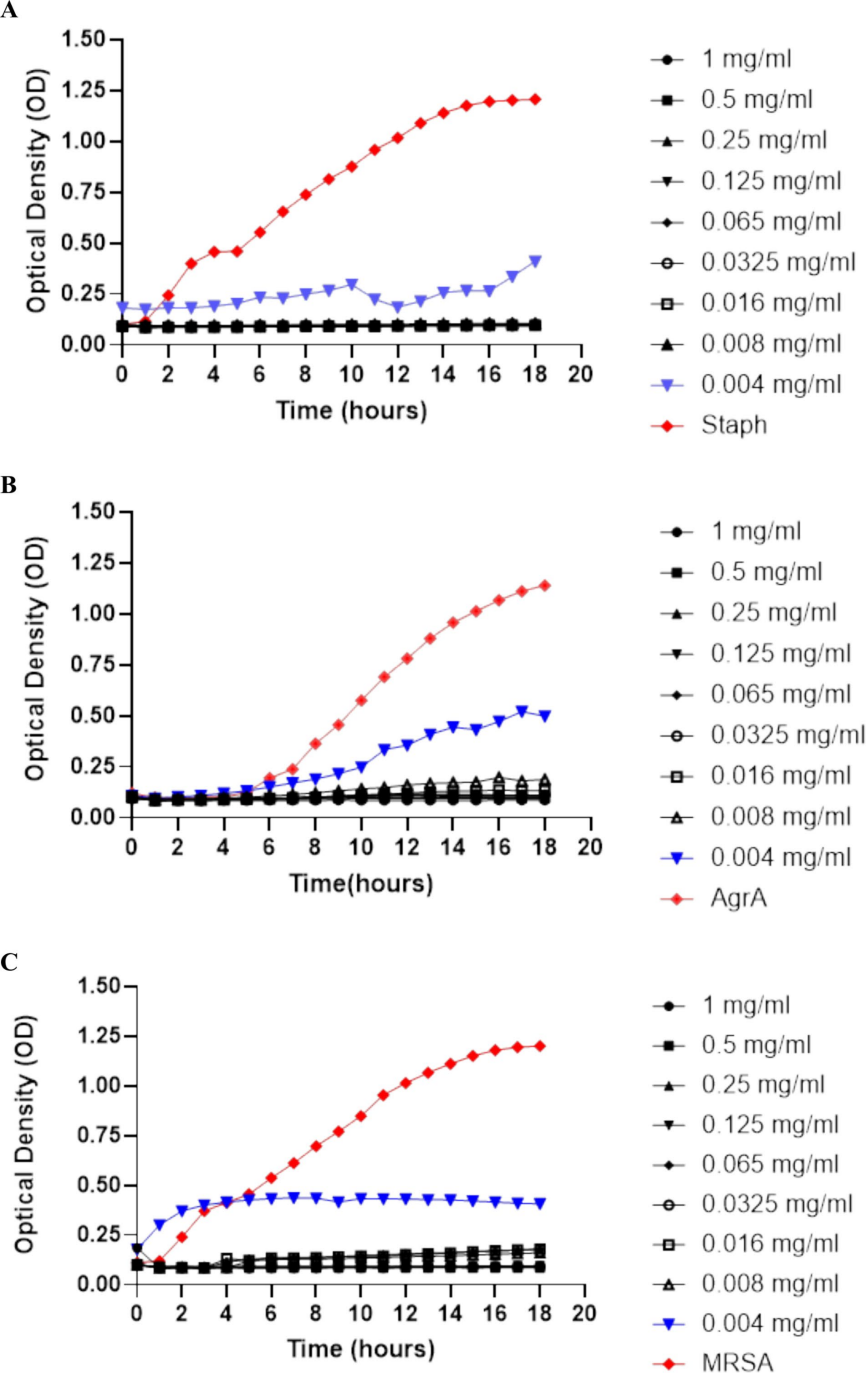
Growth curves of **(A)** *S. aureus*, **(B)** *ΔagrA*, and **(C)** MRSA were produced by measuring optical density (OD) at 600 nm over time to assess bacterial growth. The concentrations of SOL tested ranged from 1 mg/mL to 0.004 mg/mL. Concentrations of 0.5 mg/mL or higher completely suppressed growth, indicating SOL’s dose-dependent inhibitory effect on all tested strains. Data were expressed as mean ± SD (*n* = 3).

The growth curves show that SOL effectively inhibits bacterial growth in a dose-dependent manner, as demonstrated in Figure 1. Complete inhibition of growth was observed in standard *S. aureus*, MRSA, and *ΔagrA* at high concentrations (1 mg/mL and 0.5 mg/mL) with *p*-value <0.0001, indicating significant inhibiting of bacterial growth. These concentrations effectively suppressed bacterial growth throughout the duration of the experiment. However, at lower concentrations (0.065 mg/mL and below), the curves indicate a gradual increase in OD (optical density), suggesting that the bacteria were able to grow despite the presence of the antibiotic.

#### 3.1.3 Colony counting

The colony counting results showed that SOL has an antibacterial effect that increases with the concentration. At the highest concentrations (1 mg/mL and 0.5 mg/mL), no bacterial colonies were observed for *S. aureus*, MRSA, and *ΔagrA*. However, a few colonies were observed at intermediate concentrations (0.25 mg/mL and 0.125 mg/mL), and the number of colonies started to increase when the concentration of SOL was below 0.065 mg/mL.

### 3.2 Anti-virulence assays

#### 3.2.1 Hemolysis test

**Figure 2:**
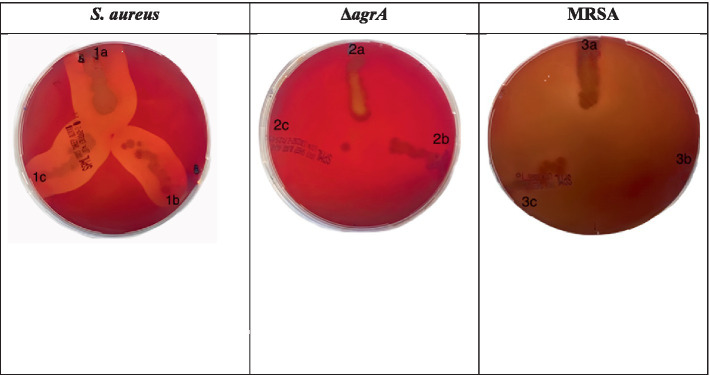
Hemolysis assays were performed on blood agar plates for (1) *S. aureus* strains, (2) Δ*agrA*, and (3) MRSA grown (a) without or with (b) inhibitory/(c) sub-inhibitory concentrations of SOL (0.8 μg/mL and 0.4 μg/mL). Zones of clearance around bacterial colonies indicate hemolytic activity.

The hemolysis assay results, illustrated in Figure 2, indicate that the wild-type *S. aureus* control exhibited a clear zone of hemolysis, unaffected by SOL treatment. The Δ*agrA* strain showed minimal hemolytic activity without SOL and none with treatment. The MRSA strain displayed no hemolytic activity in either the control or the treated conditions.

#### 3.2.2 Protease test

**Figure 3:**
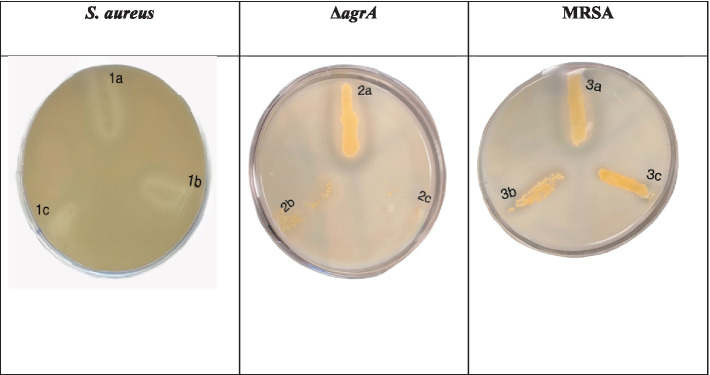
Protease activity of *S. aureus*, MRSA, and Δ*agrA* strains on skimmed milk agar plates after incubation with SOL at MIC_90_ (0.8 μg/mL) and MIC_50_ (0.4 μg/mL). Each plate represents different bacterial strains exposed to varying conditions. The clearing zone around the bacterial streaks indicates protease production and milk protein degradation.

The wild-type *S. aureus* and its variants showed minimal to no zones of clearing at MIC_50_ and MIC_90_. The Δ*agrA* strain exhibited slight protease activity with a small zone of clearing in the untreated control, while SOL treatment significantly reduced protease activity, particularly at MIC_90_. The MRSA strain displayed notable protease activity in the absence of treatment; however, the addition of SOL greatly diminished this activity, resulting in minimal or no zones of clearing at both MIC_50_ and MIC_90_. These findings indicate that SOL effectively inhibits protease production in both MRSA and Δ*agrA* strains, with a more pronounced effect (Figure 3).

#### 3.2.3 Motility test

Untreated wild-type *S. aureus* exhibited significant motility, indicated by the diffuse spread of colonies. However, treatment with SOL at both MIC_50_ and MIC_90_ reduced motility, leading to more localized colonies. The Δ*agrA* strain showed reduced motility compared to the wild type, and SOL treatment further inhibited colony spread (Palma et al., 2022). MRSA displayed moderate motility and swarming patterns, but SOL significantly restricted this motility, especially at MIC_90_. Overall, these results indicate that SOL effectively reduces motility in *S. aureus*, MRSA, and Δ*agrA* strains, particularly at MIC_90_, impairing the bacteria’s ability to spread and colonize (Rasmussen et al., 2011).

**Figure 4:**
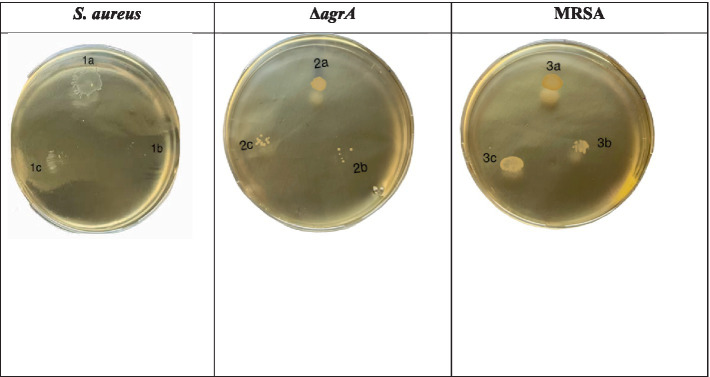
Motility assessment of *S. aureus* (1a–c), Δ*agrA* (2a–c), and MRSA (3a–c) strains on modified spreading motility media. The strains were adjusted to OD 0.5 McFarland and inoculated with or without SOL at MIC_90_ (0.8 μg/mL) and MIC_50_ (0.4 μg/mL). Plates were inoculated using a sterile cotton swab and incubated at 37°C for 24 h. Motility was determined by the extent of colony migration from the point of inoculation.

The modified spreading motility assay (Palma et al., 2022) was performed to assess the effect of SOL at MIC_50_ (0.4 μg/mL) and MIC_90_ (0.8 μg/mL) on *S. aureus*, MRSA, and *∆agrA*. In the untreated controls (1a, 2a, 3a), all strains exhibited a distinct spread from the inoculation point, indicating their ability for colony expansion on semi-solid media (Figure 4). When treated with SOL, however, the diameter of colony spread was visibly reduced, most notably at MIC_90_. This reduction suggests SOL may interfere with the bacterial mechanisms underlying spreading motility.

#### 3.2.4 Biofilm formation/inhibition assays

##### 3.2.4.1 Crystal violet assay

**Figure 5:**
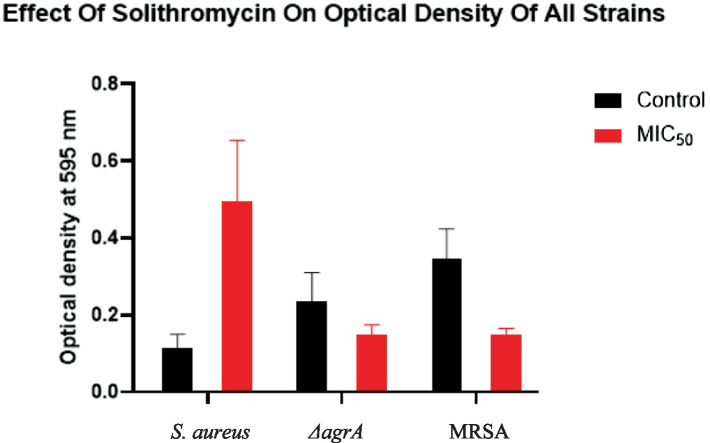
The effect of SOL on biofilm formation of *S. aureus, ΔagrA*, and MRSA strains as measured by optical density (OD) at 595 nm using the crystal violet assay. The bacterial strains were treated with SOL at MIC_50_ (0.4 μg/mL) and compared to untreated controls. Each bar represents the mean OD of the biofilm, with error bars indicating the standard deviation. Mean ± SD values: *S. aureus* (control: 0.116 ± 0.035, MIC_50_: 0.495 ± 0.159), MRSA (control: 0.237 ± 0.074, MIC_50_: 0.150 ± 0.025, *p* < 0.0001), and Δ*agrA* (control: 0.345 ± 0.078, MIC_50_: 0.150 ± 0.015, p < 0.0001).

The crystal violet assay results, presented in Figure 5, demonstrate the impact of SOL on biofilm formation in *S. aureus*, MRSA, and Δ*agrA* strains. In the untreated control groups, biofilm formation varied significantly among the strains. The Δ*agrA* strain exhibited the highest biofilm production (OD~595~ ≈ 0.5), followed by MRSA (OD~595~ ≈ 0.4), while *S. aureus* showed the lowest biofilm formation (OD ~ 595 ~ <0.2). These differences in baseline biofilm production are consistent with previous studies, which have shown that the agr system is critical in regulating biofilm formation in *S. aureus* (Goc et al., 2023; Liu et al., 2015).

Treatment with SOL at MIC_50_ (0.4 μg/mL) significantly reduced biofilm formation in all tested strains. The Δ*agrA* strain showed the most pronounced reduction, with biofilm production decreasing by approximately 90% (OD~595~ ≈ 0.05, *p* < 0.0001). Similarly, MRSA exhibited a substantial decrease in biofilm formation, reducing approximately 75% (OD~595~ ≈ 0.1, *p* < 0.0001). In contrast, *S. aureus* did not show a reduction in biofilm formation. These results suggest that SOL is highly effective at inhibiting biofilm formation in Δ*agrA* and MRSA strains, while its effect on *S. aureus* is less pronounced.

The differential response to SOL treatment may be attributed to the role of the agr system in biofilm regulation. The Δ*agrA* strain, which lacks a functional agr system, is more susceptible to SOL-mediated biofilm inhibition, likely due to the disruption of quorum-sensing pathways essential for biofilm maturation (Rodgers et al., 2013). In contrast, the wild-type *S. aureus* strain, which retains a functional agr system, may exhibit partial resistance to SOL-mediated biofilm inhibition, as the agr system can compensate for some of the inhibitory effects of SOL (Tsakou et al., 2020).

Statistical analysis using one-way ANOVA confirmed that the differences in biofilm formation between treated and untreated groups were highly significant (*p* < 0.0001 for Δ*agrA* and MRSA). These findings highlight the potent antibiofilm activity of SOL, particularly against strains with impaired *agr* function, and underscore its potential as an antivirulence agent for targeting biofilm-associated infections.

##### 3.2.4.2 Congo red agar test (CRA)

**Figure 6:**
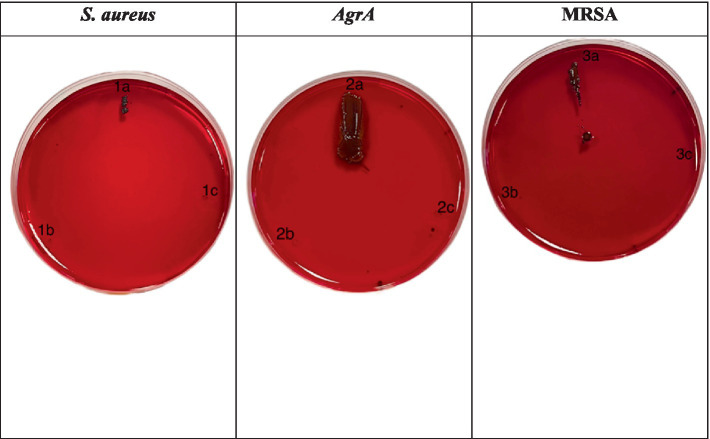
Congo red agar assay for biofilm formation by *S. aureus* (1a–c), *ΔagrA* (2a–c), and MRSA (3a–c) strains treated with SOL at MIC_50_ (0.4 μg/mL) and MIC_90_ (0.8 μg/mL). The strains were inoculated on Congo red agar plates to evaluate biofilm production. Black colonies on the red agar indicate biofilm formation, while red colonies suggest inhibition or absence of biofilm production.

The Congo red agar assay results in Figure 6 demonstrate that SOL significantly reduces biofilm formation in *S. aureus*, MRSA, and Δ*agrA* strains. In the control for *S. aureus*, distinct black colonies indicated active biofilm and slime production. Treatment with SOL at MIC_50_ partially reduced biofilm formation, while at MIC_90_, biofilm production was substantially diminished, with very few black colonies remaining. Similarly, the Δ*agrA* strain displayed dense black colonies in the control, but treatment with SOL at MIC_50_ noticeably reduced colony formation, with a dramatic decrease at MIC_90_. The control also showed biofilm formation for MRSA, but to a lesser extent. SOL at MIC_50_ resulted in smaller, lighter colonies, and at MIC_90_, biofilm formation was nearly completely inhibited. Overall, SOL effectively suppressed biofilm and slime production in all tested strains, especially at MIC_90_, highlighting its potential as a powerful agent against biofilm-forming bacteria.

##### 3.2.4.3 Scanning electron microscope analysis (SEM)

**Figure 7:**
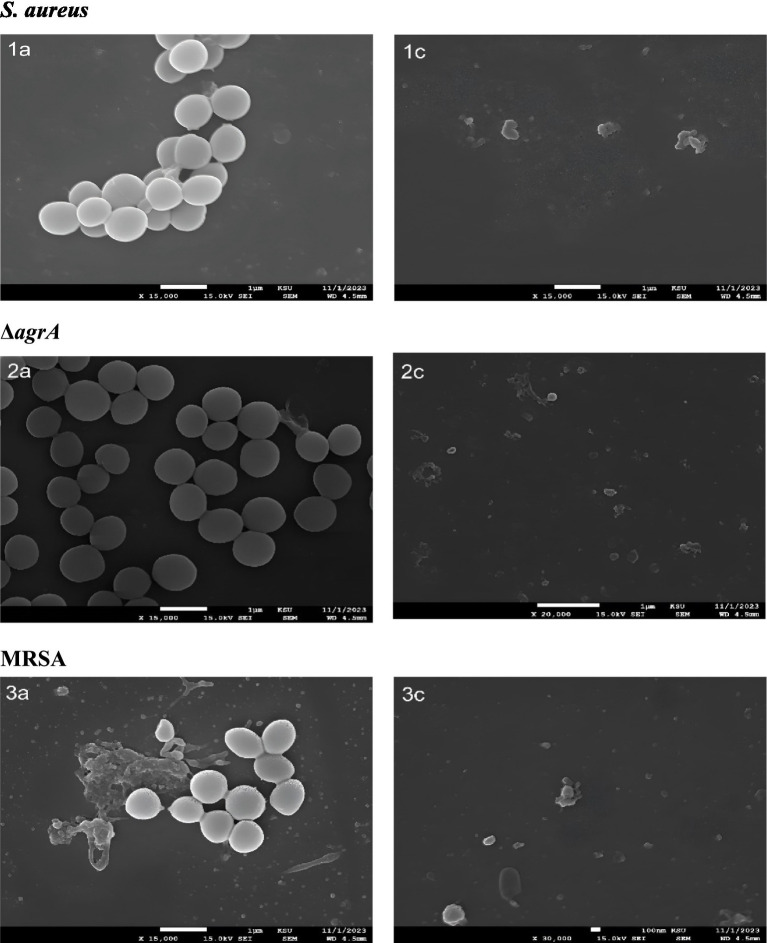
Scanning electron microscopy (SEM) analysis of *S. aureus,* Δ*agrA,* and MRSA strains treated with SOL at MIC_50_ (0.4 μg/mL) and MIC_90_ (0.8 μg/mL). The bacteria were cultured on polyvinyl coverslips in LB broth, with and without SOL to assess the morphology. After incubation, samples were fixed, dehydrated, and gold-coated for SEM imaging to assess the structural integrity of bacterial cells after treatment with SOL.

SEM revealed pronounced structural differences between untreated and SOL-treated bacteria. In untreated bacterial cultures, SEM micrographs showed dense, multi-layered clusters of cocci in an extensive extracellular matrix. In contrast, SOL-treated bacterial cultures were thin and disaggregated. The bacterial cells in treated samples appeared damaged many cells had irregular, collapsed morphologies and pitted surfaces indicative of cellular injury (Figure 7).

#### 3.2.5 Confocal laser scanning microscopy (CLSM) at MIC_50_

**Figure 8:**
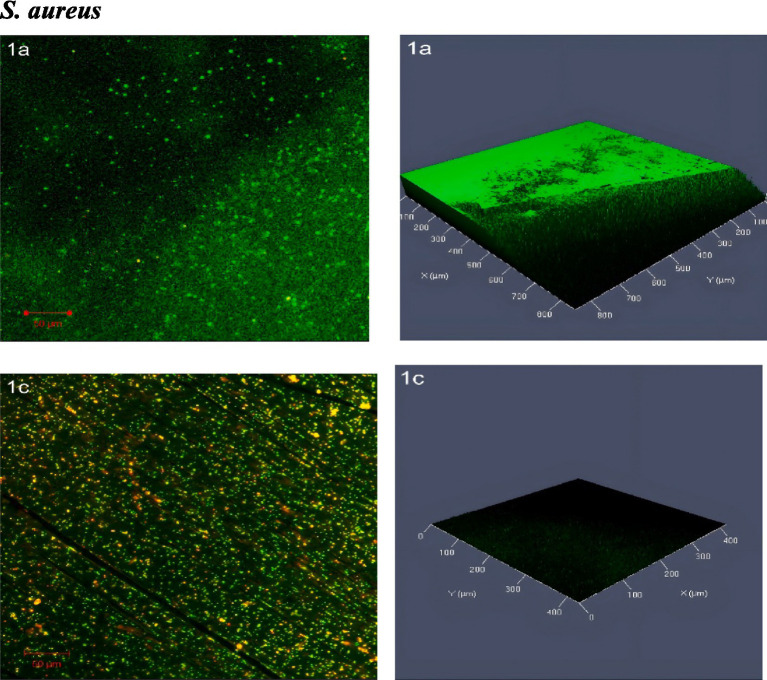
Confocal laser scanning microscopy (CLSM) analysis of (1a) *S. aureus* biofilms and (1c) treated with SOL at MIC_50_ (0.4 μg/mL). Biofilms were grown on polyvinyl coverslips in LB broth for 24 h. Live cells were stained with SYTO 9 (green fluorescence), while dead cells were stained with propidium iodide (red fluorescence). The antibiofilm potential of SOL was visualized and quantified by imaging the biofilm structures under CLSM.

CLSM images of *S. aureus* biofilms show significant differences between untreated and SOL-treated samples. The untreated biofilm displayed a dense structure with a high concentration of live cells, indicated by green fluorescence, and very few dead cells marked by red fluorescence, suggesting a healthy biofilm. In contrast, treatment with SOL at MIC_50_ (0.4 μg/mL) disrupted the biofilm, reducing live cell density and increasing dead cells, highlighting the antibiofilm activity of SOL (Figure 8).

**Figure 9:**
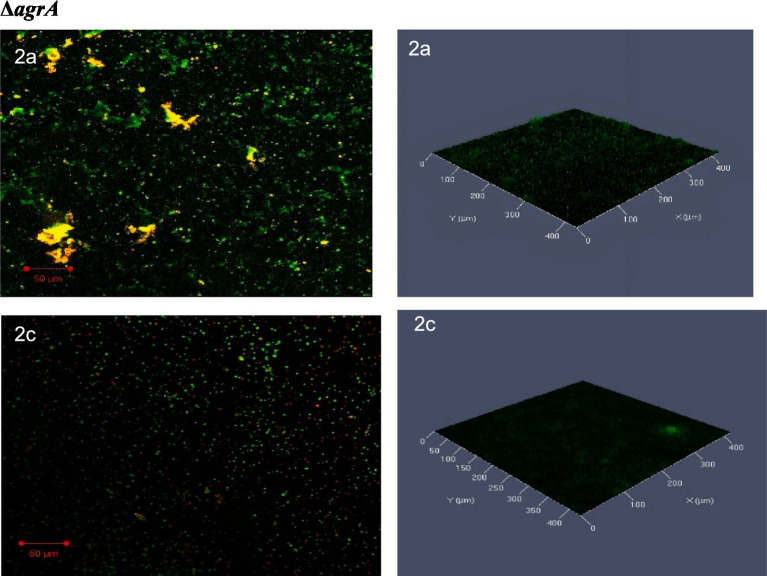
Confocal laser scanning microscopy (CLSM) analysis of (2a) *ΔagrA* biofilms, and (2c) treated with SOL at MIC_50_ (0.4 μg/mL). Biofilms were developed on protein-coated polyvinyl coverslips in LB broth for 24 h. Live cells were stained with SYTO 9 (green fluorescence), while dead cells were stained with propidium iodide (red fluorescence). The biofilm structures were visualized to assess the impact of SOL on biofilm formation and bacterial viability.

The confocal laser scanning microscopy (CLSM) images of the *ΔagrA* biofilms revealed significant differences between the untreated and SOL-treated samples. In the untreated biofilm, the *ΔagrA* strain formed a thick, well-organized biofilm, characterized by abundant live cells (green fluorescence) and minimal dead cells (red fluorescence), indicating a strong biofilm structure with high bacterial viability. Following SOL treatment at MIC_50_ (0.4 μg/mL), the biofilm structure showed clear signs of disruption, with a noticeable reduction in green fluorescence, reflecting a decrease in live cell numbers, and an increase in red fluorescence, indicating more dead cells. Although the biofilm structure remained intact, its density was reduced (Figure 9).

**Figure 10:**
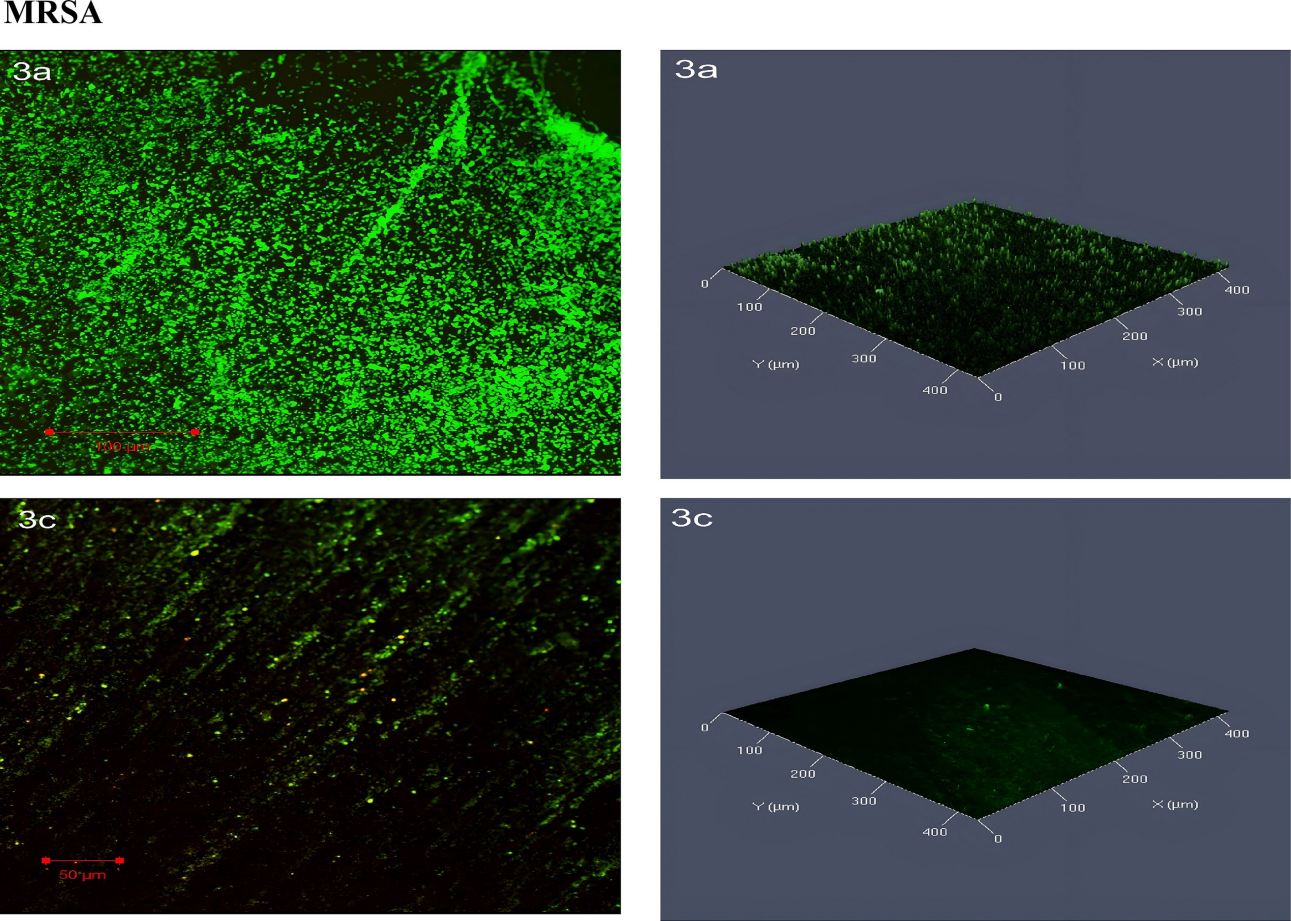
Confocal laser scanning microscopy (CLSM) analysis of (3a) MRSA biofilms, and (3c) MRSA treated with SOL at MIC_50_ (0.4 μg/mL). Biofilms were grown on protein-coated polyvinyl coverslips in LB broth for 24 h. Live cells were stained with SYTO 9 (green fluorescence), and dead cells were stained with propidium iodide (red fluorescence). The antibiofilm and bactericidal potential of SOL were visualized through CLSM imaging.

The confocal laser scanning microscopy (CLSM) images of MRSA biofilms revealed distinct differences between untreated and SOL-treated groups. In the untreated biofilm, the MRSA cells were densely packed, with strong green fluorescence indicating a high concentration of live bacterial cells. Very few dead cells were present, as shown by sparse red fluorescence, suggesting that the untreated biofilm are largely viable and structurally intact. After treatment with SOL at MIC_50_ (0.4 μg/mL), the biofilm became more dispersed, and a noticeable increase in red-stained dead cells was observed. Although some live cells remained, the overall biofilm density was reduced, and the structure was less cohesive, indicating a partial antibiofilm effect (Figure 10).

**Figure 11:**
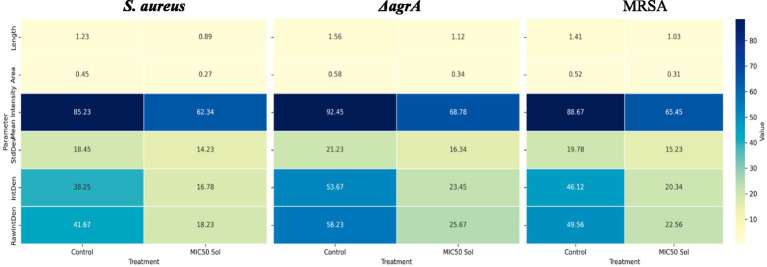
Heatmap visualization of quantitative parameters derived from confocal laser scanning microscopy (CLSM) and analyzed using ImageJ software.

The analysis compares untreated controls with MIC_50_ SOL-treated biofilms across three bacterial strains: *S. aureus*, Δ*agrA* mutant, and MRSA. Six key parameters were measured (Figure 11):

- Length: Physical dimension of bacterial clusters (measured in μm).
- Area: Surface coverage of bacterial biofilm (measured in μm^2^).
- Mean Intensity: Average fluorescence intensity, indicating biomass density.
- StdDev: Standard deviation of fluorescence intensity, representing biofilm heterogeneity.
- IntDen: Integrated density, measuring total fluorescence signal.
- RawIntDen: Raw integrated density, representing absolute fluorescence values.

The heatmaps show numerical values for each parameter (displayed within cells) using a yellow-green-blue color gradient, where lighter colors indicate lower values and darker colors denote higher values. All measurements were conducted in triplicate, with mean values displayed.

Figures 7, 8 shows that all strains exhibited reduced values across all parameters following MIC50 Sol treatment. *ΔagrA* showed the highest baseline values in control conditions. The most significant reductions were observed in the IntDen and RawIntDen parameters. MRSA displayed intermediate susceptibility to Sol treatment compared to wild-type *S. aureus* and *ΔagrA.* These quantitative measurements demonstrate the effectiveness of SOL in diminishing biofilm formation across different *S. aureus* strains, with varying degrees of susceptibility observed between wild-type and mutant strains. Values represent means from three independent experiments (*n* = 3). Statistical significance was determined using Student’s t-test, with *p* < 0.05 considered significant.

### 3.3 Molecular assays

#### 3.3.1 Gene detection using conventional PCR

**Figure 12:**
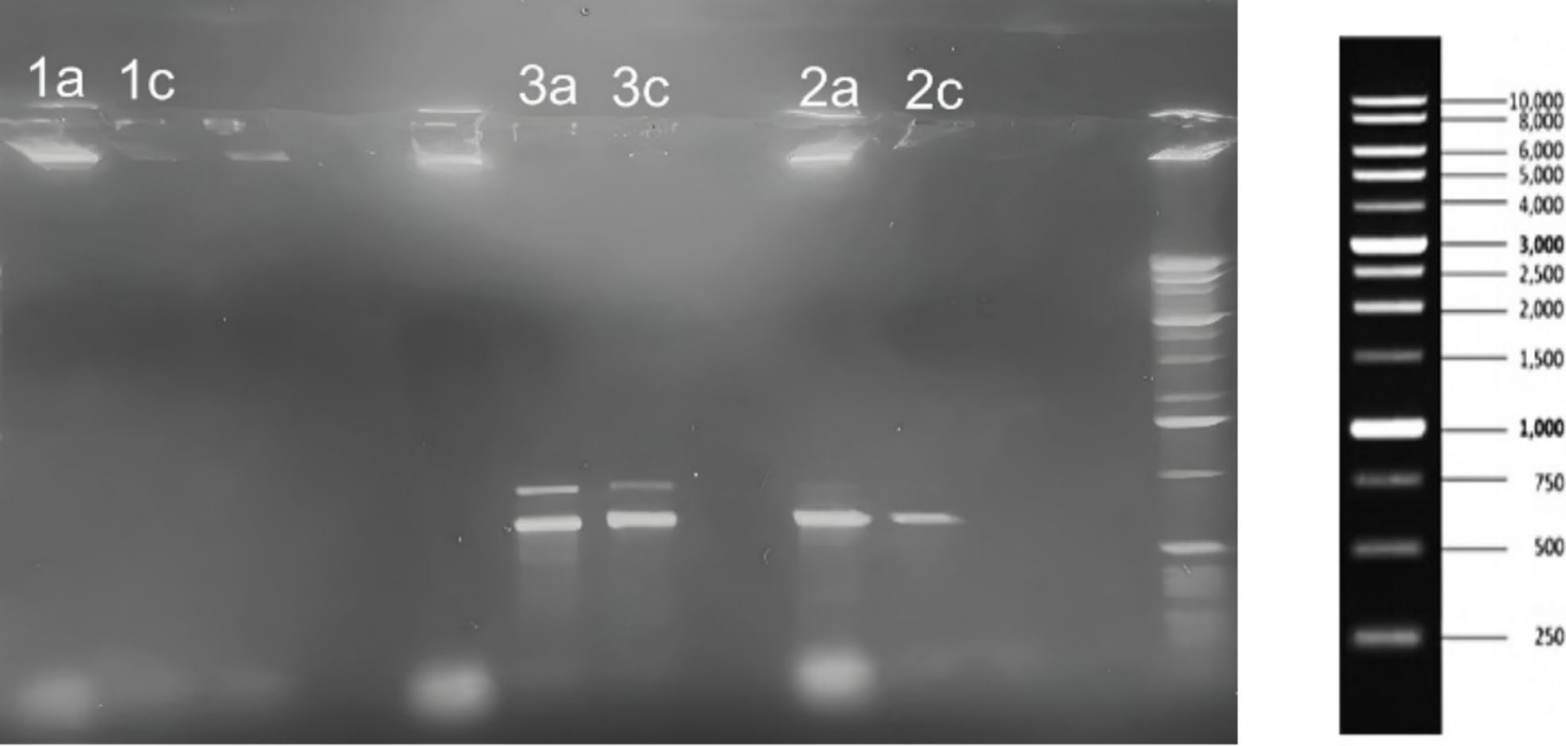
Agarose gel electrophoresis results showing PCR amplification of splB (720 bp), hla (879 bp), and lukF (930 bp) genes from *S. aureus* (1a, 1c), MRSA (3a, 3c), and *ΔagrA* (2a, 2c) strains treated with SOL at MIC_50_ (0.4 μg/mL). DNA was extracted, amplified by PCR, and separated on a 1% agarose gel. The gel included a 1 Kb DNA ladder (right) to indicate the size of the amplified gene products.

Agarose gel electrophoresis was used to qualitatively assess the presence of key virulence genes after SOL treatment. Figure 9 shows PCR amplification of the *splB* (720 bp), *hla* (879 bp), and *lukF-PV* (930 bp) gene targets for the wild-type *S. aureus*, MRSA, and Δ*agrA* strains, with and without SOL (MIC_50_) treatment. The PCR results suggested the presence of the *splB* (protease) and *hla* (alpha-hemolysin) genes in the wild-type and MRSA strains. As still need to be confirmed, the *ΔagrA* mutant lacked an *hla* band (consistent with agrA’s role in regulating *hla* expression), but showed a band for *splB*. The leukocidin gene *lukF-PV* was not detected in any of the strains under our experimental conditions (Figure 12).

These results are presented as preliminary, qualitative observations. We have refrained from making definitive conclusions about gene expression levels from band intensity alone. To conclusively determine if SOL at sub-inhibitory concentrations down-regulates virulence gene expression, quantitative methods such as RT-qPCR would be required.

## 4 Discussion

The findings from this study establish solithromycin (SOL), a next-generation macrolide, as an antivirulence agent, targeting selected virulence factors of *S. aureus*, including MRSA and *ΔagrA* strains. The results are, therefore, in agreement with the hypothesis that SOL selectively inhibits biofilm formation and motility—essential mechanisms for the persistence and dissemination of infection—while sparing other mechanisms of virulence. The targeted approach is consistent with our research question, which asked if SOL could act as an antivirulence strategy, whereby factors contributing to bacterial survival and colonization are targeted without bactericidal effects that are often associated with the development of resistance (Fleitas Martínez et al., 2019).

The antibacterial assays conducted in the study demonstrated that SOL significantly inhibited the growth of *S. aureus*, MRSA, and *ΔagrA* strains at both MIC_90_ and MIC_50_ concentrations. The clear zones of inhibition observed in the agar diffusion test, alongside the complete suppression of bacterial growth at higher SOL concentrations in the growth curve assays, underscore SOL’s potent antibacterial activity. These results agreed with previous studies indicating SOL’s efficacy against various bacterial pathogens, including those resistant to other macrolides (Farrell et al., 2016). However, the gradual increase in optical density (OD) at lower concentrations suggests that while SOL is effective, its potency reduces as the concentration decreases. This observation is consistent with the typical behavior of antimicrobial agents, where efficacy is concentration-dependent (Still et al., 2011). The colony-counting results corroborate the growth curve findings, with no colonies observed at the highest concentrations of SOL.

There have been previous studies on SOL’s antimicrobial activity against *S. aureus* (Yao et al., 2019). Our findings expand on their work by specifically examining its impact on hemolytic activity. Although MRSA *ΔagrA* showed minimal hemolytic activity, the hemolysis test results indicate that SOL does not affect hemolytic activity in wild-type *S. aureus*.

The protease assay shows SOL’s ability to inhibit virulence factor production. The significant reduction in protease activity, especially in MRSA and *ΔagrA*, indicates that SOL interferes with the bacterial mechanisms responsible for protease production. The impact observed on the Δ*agrA* strain is particularly interesting, as the agr system is a key regulator of virulence in *S. aureus*. SOL affects protease activity in this strain, suggesting that its mechanism of action may involve pathways independent of or downstream from the agr system (Fleitas Martínez et al., 2019). The motility test results indicate that SOL significantly impairs bacterial motility across all tested strains. The reduction in colony spread at MIC_50_ and MIC_90_ concentrations suggests that SOL may inhibit the motility machinery of *S. aureus*, MRSA, and Δ*agrA*. While *S. aureus* is not typically considered a highly motile organism compared to flagellated bacteria, it does exhibit forms of motility such as spreading and gliding, which are crucial for colonization and biofilm formation (Shen et al., 2020). This motility is regulated by the agr system, which controls the production of surfactant peptides, essential for surface colonization. Research indicates that agr knockout mutants cannot effectively spread, showcasing the importance of this system in establishing infections (Shen et al., 2020).

SOL’s impact on motility suggests it may reduce *S. aureus* colonization and the spread of infections by hindering its movement across surfaces. A non-motile *S. aureus* would be less capable of forming biofilms and migrating, ultimately decreasing its virulence. Thus, SOL’s effects on motility complement its antivirulence strategy, may contribute to biofilm suppression and toxin regulation (Shen et al., 2020).

The findings on SOL’s disruptive action on biofilm formation highlight its potential as an antivirulence agent against *S. aureus*. Biofilms in bacterial pathogens such as *S. aureus* produce complex microbial communities that greatly enhance bacterial resistance both to antimicrobial therapies and to host immune responses. Such resistance confounds infection management, especially in hospital settings or within indwelling medical devices where biofilm formation often arises. The agr quorum-sensing system is a central regulator of biofilm formation in *S. aureus*, controlling the expression of genes responsible for the production of extracellular polysaccharide (EPS), which represents the biofilm matrix and enhances its stability (Taj and Chattopadhyay, 2024).

SOL’s ability to interfere with biofilm development seems to be associated with its influence on the agr quorum-sensing pathway, which is a main modulator of genes associated with biofilms. While the agr system regulates many factors that are involved in the structure and maintenance of biofilms, dysfunction in the agr may further cause a decline in the expression of its target genes responsible for the promotion of biofilms, consequently weakening the biofilm matrix and thus increasing the susceptibility of cells to different environmental stresses (Le and Otto, 2015). This approach aligns with the growing interest in antivirulence strategies as alternatives to traditional antibiotics, especially in the face of increasing antibiotic resistance (Dickey et al., 2017). In this study, the effects of SOL on the biofilm formation were confirmed as demonstrated by the crystal violet and Congo red agar assays. The reduced biofilm mass in these assays suggests that SOL can directly inhibit agr-mediated signaling, impairing EPS synthesis and reducing the maturation of biofilms. Similar observations have been reported in other macrolides, for instance in the study by Amábile-Cuevas (2023), which observed that macrolides affect quorum-sensing pathways, further inhibiting biofilm formation without lethal effects on bacteria. SOL acts as a highly valued anti-virulence agent since it can inhibit *S. aureus* biofilm formation.

SOL showed that it could prevent biofilm formation at both the MIC_50_ and MIC_90_ values of 0.4 μg/mL and 0.8 μg/mL, respectively. The antibacterial assay was extraordinary and suggested that either its structure or mode of action was different from typical macrolides in a way that it could not be subjected to the compensatory biofilm formation usually stimulated by sub-inhibitory antibiotic exposure. The study on chemical inhibition of the agr quorum-sensing system in *S. aureus* identified targeting of this pathway as consistently resulting in the reduced ability to form biofilms (Dickey et al., 2017). The agr system is repressing the production of EPS required for the stability of the biofilm. Biofilm inhibition through SOL likely results from its inhibition of agr signaling in a manner analogous to targeted inhibition by quorum-sensing inhibitors, but it indicated the broader efficacy of SOL against multiple strains of *S. aureus*, including MRSA.

Similarly, the research by Taj and Chattopadhyay (2024) on components of the *S. aureus* biofilm also brings to light the role of EPS in the robustness of biofilms and adherence of bacteria via the agr system. The source underlines the efficiency of targeting agr for the downregulation of EPS synthesis—a concept similarly shared by SOL in our research, as presented by the reduced production of EPS in the Congo red agar tests. This effect on EPS production differentiates SOL from other macrolides, which generally do not disrupt the synthesis of EPS. Thus, the effect of SOL on EPS, a major component of biofilm, seems to present a more comprehensive approach to biofilm inhibition.

SEM and CLSM imaging provided insights into structural effects induced by SOL in the biofilms. The untreated biofilms were thick and multilayered, representative of healthy biofilm structures. On the other hand, SEM images for the SOL-treated samples showed thin and dispersed bacterial colonies as evidence of loss of integrity in these biofilms (see Figure 10). This was further supported through the observation of CLSM images showing increased red fluorescence, indicative of dead cells and thus a compromised biofilm matrix, within the SOL-treated biofilms (see Figure 11). Such structural disruptions are most probably the reflection of the effect of SOL on the pathways of cellular communication that are crucial for the coherence of the biofilms. This technique has been successfully applied in various studies to assess the antimicrobial effects of drugs on biofilms, enabling researchers to discriminate between live and dead bacterial cells and localize their spatial distribution within the biofilm (Relucenti et al., 2021).

The presence of distinct PCR product bands for *splB* and *hla* genes in MRSA, and only *splB* in the Δ*agrA* strain, suggests that SOL may have varying effects on gene expression depending on the specific strain and its genetic background. This observation aligns with previous studies that have shown differential effects of antibiotics on virulence gene expression in *S. aureus* (Ren et al., 2022). The reduced band intensity observed in the SOL-treated samples compared to untreated controls may indicates that SOL at MIC_50_ reduce gene expression or bacterial activity. The absence of *hla* represented band in the Δ*agrA* strain is consistent with previous studies showing that the agr system positively regulates alpha-hemolysin production (Ren et al., 2022). The observation that *splB* is detected in the Δ*agrA* strain suggests that this gene may be regulated by additional factors beyond the agr system. This finding underscores the complexity of virulence regulation in *S. aureus* and the potential for strain-specific responses to antibiotic treatment. The reduced expression of virulence genes in response to SOL treatment at sub-inhibitory concentrations (MIC_50_) is particularly noteworthy (Figure 12).

In this study, the *ΔagrA* strain—deficient in the agrA response regulator—was included to clarify whether SOL exerts its antivirulence activity solely through the agr quorum-sensing pathway or through additional mechanisms. As shown in Section 3.2.1 (Hemolysis), and Section 3.2.2 confirmed that *ΔagrA’*s baseline protease activity was lower than that of the wild-type strain, yet it remained substantially inhibited by SOL, indicating agr-independent suppression of certain virulence factors.

We also observed in Section 3.3.3 (Motility) that *ΔagrA* exhibited restricted colony spreading compared to wild-type *S. aureus*. Most notably, the biofilm results in Section 3.4 showed that despite *ΔagrA* typically forming robust biofilms. SOL still significantly disrupted *ΔagrA* biofilm formation. This partial inhibition suggests that SOL can modulate pathways beyond agr for biofilm regulation.

Together, these findings indicate that while agr-mediated signaling is a major driver of virulence in *S. aureus*, solithromycin’s antivirulence effects extend beyond the AgrA regulon. This observation supports prior work showing that sub-inhibitory macrolide or ketolide concentrations can downregulate *S. aureus* virulence factors through broader translational interference.

Consequently, the *ΔagrA* strain provided a crucial means to distinguish agr-dependent effects from other potential mechanisms by which SOL attenuates pathogenic traits, shedding light on its multifaceted role in suppressing staphylococcal virulence (Sun et al., 2012).

SOL is known to bind the 50S ribosomal subunit, thereby inhibiting protein synthesis. This disruption of translational processes can reduce the production of virulence factors in *S. aureus* at bacteriostatic or sub-inhibitory concentrations. Indeed, the partial inhibition of the *ΔagrA* mutant’s biofilm and motility traits, despite the absence of the master virulence regulator AgrA, indicates additional translational-level mechanisms. SOL may broadly impact the expression of multiple regulatory and structural genes required for pathogenesis, including those outside the agr pathway. Notably, our PCR-based assays, while suggesting decreased target gene amplification, do not definitively prove downregulation at the transcriptional level—further qPCR or transcriptomic analyses would be needed to confirm these observations. Nevertheless, the consistent phenotypic reductions in hemolysis, proteolysis, and biofilm formation support that SOL’s mechanism of action diminishes virulence by limiting the synthesis of key factors essential for *S. aureus* pathogenicity. To confirm that these effects translate to genuine transcriptional changes, quantitative RT-PCR or transcriptomic profiling would be required. Future studies in our lab will adopt these approaches to ascertain whether the observed PCR band differences indeed correlate with altered mRNA levels and overall virulence phenotypes.

## 5 Conclusion

Solithromycin’s role as an antivirulence agent against *S. aureus* and resistant variants represents a significant step forward in addressing the challenges posed by antibiotic resistance. SOL offers a novel therapeutic approach that could complement existing antibiotic therapies and contribute to more effective management of bacterial infections, via selective targeting of virulence factors critical for bacterial persistence and dissemination. The potential of SOL to reduce the selective pressure for resistance development further underscores its value as a strategic tool in the fight against antibiotic-resistant pathogens. As research continues, SOL’s integration into clinical practice could pave the way for innovative treatment strategies that list the disruption of bacterial virulence mechanisms, ultimately improving patient outcomes and mitigating the global health threat posed by antibiotic-resistant infections (Bustin et al., 2009).

## Data Availability

The original contributions presented in the study are included in the article/supplementary material, further inquiries can be directed to the corresponding author.
